# Strategies in ‘snake venomics’ aiming at an integrative view of compositional, functional, and immunological characteristics of venoms

**DOI:** 10.1186/s40409-017-0117-8

**Published:** 2017-04-28

**Authors:** Bruno Lomonte, Juan J. Calvete

**Affiliations:** 1 Instituto Clodomiro Picado, Facultad de Microbiología, Universidad de Costa Rica, San José, 11501 Costa Rica; 2 Structural and Functional Venomics Laboratory, Instituto de Biomedicina de Valencia, CSIC, Valencia, Spain

**Keywords:** Snake venoms, Proteomics, Venomics, Antivenomics, Toxicovenomics

## Abstract

This work offers a general overview on the evolving strategies for the proteomic analysis of snake venoms, and discusses how these may be combined through diverse experimental approaches with the goal of achieving a more comprehensive knowledge on the compositional, toxic, and immunological characteristics of venoms. Some recent developments in this field are summarized, highlighting how strategies have evolved from the mere cataloguing of venom components (proteomics/venomics), to a broader exploration of their immunological (antivenomics) and functional (toxicovenomics) characteristics. Altogether, the combination of these complementary strategies is helping to build a wider, more integrative view of the life-threatening protein cocktails produced by venomous snakes, responsible for thousands of deaths every year.

## Background

The potent harmful effects of snake venoms have intrigued mankind for centuries, inspiring in many cultures both fear and fascination [1]. With the advent of modern science, research on snake venoms has mainly targeted three goals [2–4]: (a) deciphering their biochemical compositions, (b) understanding their mechanisms of action and potential uses thereof, and (c) devising antidotes for the treatment of envenomation.

Snake venoms are secretions produced by a pair of specialized exocrine glands, predominantly composed by diverse peptides and proteins, many of which are endowed with enzymatic activities [5, 6]. Most of the current knowledge on venoms has been gathered by conventional biochemical and pharmacological approaches, where particular toxins are first isolated, and then studied in depth to determine their fundamental structural and mechanistic features. As expected, available information is biased towards toxins that are abundant in venoms from the most common snake species of medical relevance, leaving those of species that are scarce, or more difficult to collect and keep captive, largely unexplored.

Following the general trends in biosciences, a new era in the characterization of snake venoms began with the introduction of proteomics and related -omics technological tools, which have steered a major and rapid expansion of knowledge on their overall composition. Venoms from a growing number of snake species have been, and are being, characterized worldwide by proteomic approaches, providing an unprecedented data platform to enhance our understanding on these fascinating, but dangerous, toxic cocktails. Given that envenomation is a relevant cause of morbidity and mortality in the rural tropics of the world [7, 8], new knowledge on the biochemical constitution of venoms is of high potential impact in medicine, as discussed in the following sections. In addition, omics-based characterization of venoms is unveiling new paths to analyze fundamental questions in biology [9]. The recruitment of genes and evolution of toxic functionalities from ancestral ‘physiological’ protein scaffolds, for example, is an area of research largely powered by the recent introduction of -omic techniques to the study of snake venoms [10–13].

This work offers a general view on the evolving strategies for the proteomic analysis of snake venoms, and discusses how these may be combined with diverse experimental approaches with the goal of achieving a more comprehensive knowledge on the compositional, toxic, and immunological characteristics of venoms.

## Proteomic approaches, *pro et contra*

It is commonly said that there is no ‘one-size-fits-all’ among the various analytical strategies available for exploring the proteome of complex biological samples, since each approach has its particular advantages and disadvantages. Several reviews have previously dealt with the description of different workflows for proteomic characterization of snake venoms [14–18]. Therefore, we do not aim to present here a detailed view of their technical aspects. Rather, we highlight some of the most notable differences, *pro et contra*, among them and discuss their potential for combination with complementary methods that may expand the informative value of the datasets obtained, in terms of their biological and biomedical significance.

**Fig. 1:**
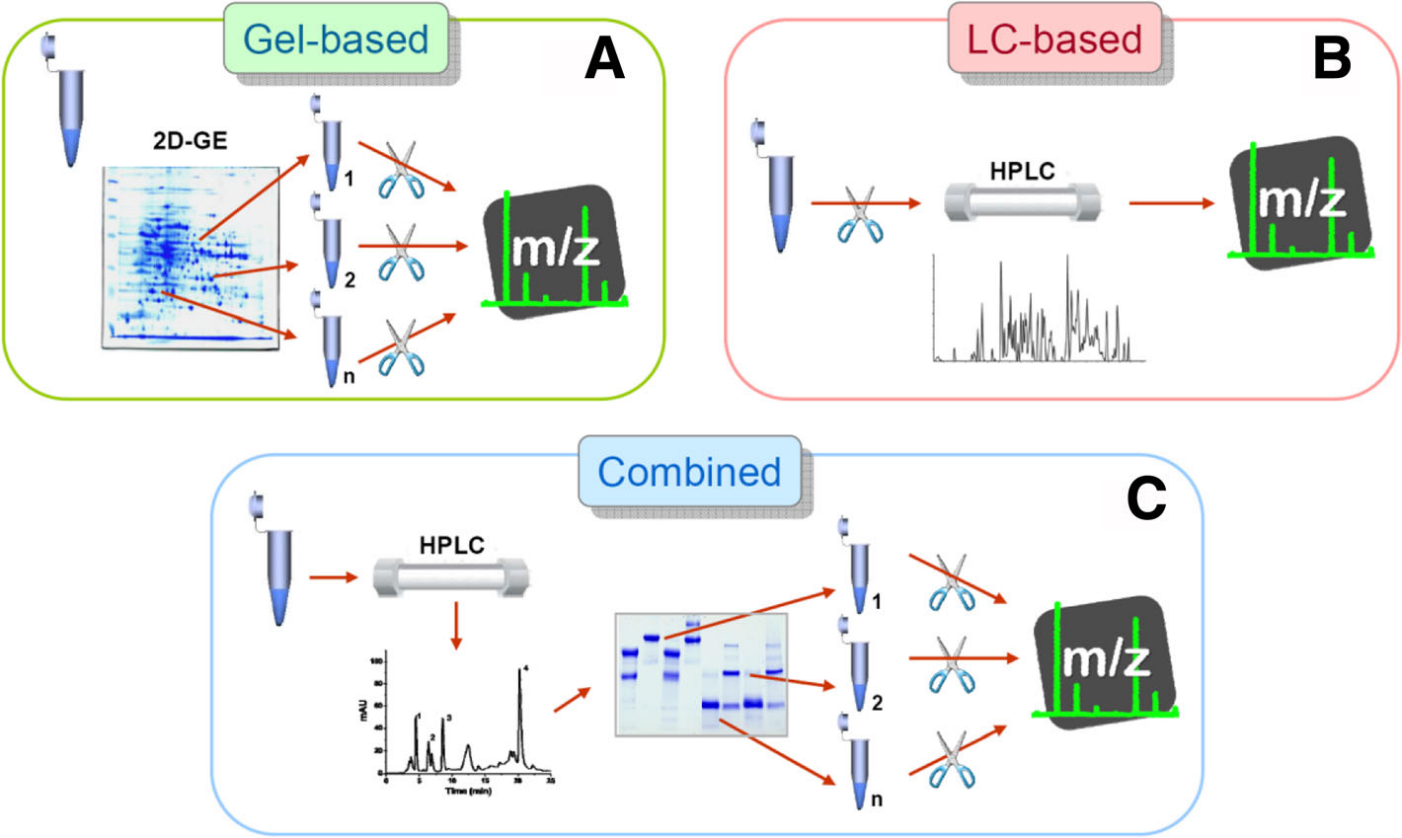
General types of analytical bottom-up strategies employed in the proteomic profiling of snake venoms. **a** Gel-based strategies involve the separation of the venom proteins by two-dimensional gel electrophoresis (2DE) followed by staining and spot picking. Protein spots are then in-gel digested (usually with trypsin, scissors icon) and the resulting proteolytic peptides submitted to tandem mass spectrometry (MS/MS) analysis. **b** Liquid-chromatography (LC)-based strategies (shotgun proteomics) digest the whole venom with trypsin and separate the resulting peptides usually by multidimensional nano-flow HPLC, hyphenated to MS/MS analysis. **c** The combined strategy of ‘snake venomics’ takes advantage of the opportunity of performing the fractionation and the quantification of the venom components in the same reversed-phase chromatography step. A second step of separation and quantification is performed by SDS-PAGE followed by gel densitometry. Protein bands are excised, in-gel digested with trypsin, and submitted to MS/MS analysis

Snake venom proteomes have been analyzed using essentially three decomplexation strategies: (a) two-dimensional gel electrophoresis (2DE)-based, (b) liquid chromatography (LC)-based, and (c) combined (LC + 1DE)-based, as schematically represented in Fig. 1. While all of these approaches converge in their goal of obtaining a catalogue, as comprehensive as technically possible, of the protein/peptide constituents of a given venom, there are differences in the overall information that can be obtained, such as the possibility of complementing the final qualitative information with an estimation of relative abundances for the venom components, or other relevant characteristics. A shared limitation of proteomic experiments dealing with any of the above-mentioned strategies is the paucity of genomic/transcriptomic databases for venomous snakes. This situation often restrains the prospect of identifying individual components, leaving only the possibility to assign them to known protein families on the basis of similarity with existing sequence entries [19]. Nevertheless, such limitation has been tackled by performing transcriptomic analyses of venom glands in combination with the proteomic profiling of venom [19–22]. This greatly enhances the performance of matching algorithms for high-resolution mass spectra and allows to move from a protein-family resolution, to a protein-locus resolution [17]. In addition to the growth of transcriptomic data, new genomic sequencing data increasingly reported for venomous snakes [23, 24] will also facilitate protein identification by automated mass spectrometry (MS) processing software.

### Gel-based proteomic strategies

Gel-based approaches (Fig. 1a) have been used in several proteomic studies on snake venoms, including some of the first reported examples [25–30]. Individual spots are excised, in-gel digested, and submitted to tandem mass spectrometry (MS/MS) analysis. Among advantages, a full pattern of sample decomplexation can be obtained in a single two-dimensional gel electrophoresis (2DE), from which information on the isoelectric point (pI, first dimension) and apparent molecular weight (Mw, second dimension) of the proteins can be readily determined for each spot. Moreover, the macromolecular organization of venom proteins can also be assessed by comparing 2DE separations run under non-reducing conditions in both directions versus non-reducing (first dimension)/reducing (second dimension) [31]. Also, it is possible to stain the gel not only for proteins, but also for conjugated moieties such as glycosylations or other post-translational modifications (PTMs) of interest [32, 33]. Furthermore, proteins can be electrophoretically transferred from the gels to membranes for subsequent immunoblotting analysis using antivenoms [29, 30, 34].

On the other hand, although 2DE analysis presumably reflects better the venom protein complexity in a single image than any other protein separation approach, limitations inherent to the gel-based strategies for proteomic profiling have also been pinpointed. First, only proteins and large peptides are retained in the electrophoretic gels, while peptides smaller than 2-3 kDa are lost. Short peptides can be abundant components of some snake venoms, and may display relevant bioactivities [35]. An additional drawback of the gel-based strategies is the limited dynamic range of protein concentrations in the original sample that can be resolved electrophoretically into non-overlapping spots, which also bears a relationship to the maximal limits in sample loads of the 2DE technique. Finally, some proteins exhibiting extreme pI’s, close to the limits of the pH gradient used in the first dimension isoelectrofocusing step, or unstable proteins with a tendency to aggregate or precipitate, may be lost, or produce inconvenient ‘streakings’ that affect the overall resolution. It is also possible that single spots might contain two or more proteins, and this is particularly evident when MS/MS identification is performed on high-end, sensitive instruments. Regarding the estimation of protein abundance, 2DE images can in principle be analyzed by densitometry. However, such quantitation can be complex, and is generally considered less reliable in comparison to the simpler band patterns generated by one-dimensional electrophoresis [17].

### LC-based proteomic strategies

LC-based proteomic profiling strategies (Fig. 1b) rely completely on the chromatographic separation of peptides resulting from the proteolytic digestion of the whole venom sample. Also known as ‘shotgun’ proteomics, in this kind of approach an impressive resolution of peptides can be obtained by reverse-phase HPLC columns at the nano-flow scale, especially when combined in-line with additional ion-exchange or other types of LC media in so-called ‘2D-LC’ or multidimensional separations. Although these strategies are well developed to provide a deep cataloguing of the protein/peptide components of the venom, the relationship of the identified peptides to their intact parent molecules is essentially lost, or very difficult to reconstruct, owing to the fact that digestion is performed on the crude venom sample as a whole. Consequently, conversion of the obtained qualitative data into a quantitative estimation of protein abundances becomes complicated.

Current high-end MS instruments and specialized software allow for ‘label-free’ (i.e., not depending on the use of isotope labeling) quantitation of peptides resolved by the nano-LC separation, based on principles such as spectral counting or peak signal integration. However, this type of quantitation is especially suited for relative comparisons of identical components among different samples, rather than for absolute estimations within a sample [36]. The fact that different peptides intrinsically present large variations in their ionization efficiency is an obvious obstacle for absolute abundance estimations. Furthermore, factors such as the multidomain construction of some snake venom protein families (e.g., metalloproteinases, multimeric complexes, etc.) introduce uncertainties in the assignment of tryptic peptides to intact parent molecules if these are digested together.

On the other hand, some features of the LC-based strategies make them an attractive option for the study of snake venoms, such as the simple preparation of samples, and the high-speed/high-throughput, automated processing of the LC-MS/MS runs, together with the deep detection of trace protein components. Notwithstanding, these powerful strategies have thus far provided most often qualitative information on venom composition. It should be stressed that relative protein abundances reported in some studies based on this analytical pipeline [37, 38], as well as on the 2DE workflow [39, 40], correspond to ‘frequency of identification’, or ‘percentage of the protein sequences’, which may not be necessarily equivalent to abundance [41], and may therefore not reflect the actual quantitative distribution of components in the venom. Thus, in all peptide-based quantitation techniques, the assumption is made that protein digestion is complete, and that the resulting proteolytic peptides are equally detectable by the mass spectrometric technique used for the analysis.

In addition, the assumption ‘one peptide = one protein’ is obviously not true for proteins with repeat units, or for highly similar isoforms that share large parts of their amino acid sequences. Moreover, shotgun strategies do not allow further combinations with appended techniques to expand the informative value of the analyses. Further, owing to the fully automated processing of matching the fragmentation spectra against databases, limitations on available information for snake proteins become of concern. New algorithms for proteomic analysis are achieving impressive progress and efficiency in the automated *de novo* sequencing of peptides from MS/MS spectra [42–44], and this may counterbalance the problem of venom proteins database limitations.

### Combined LC/gel-based proteomic strategies

A workflow combining an LC first dimension separation, with a one-dimensional electrophoresis (SDS-PAGE) as second dimension, was introduced by Calvete et al. [45, 46] who referred to it as ‘snake venomics’. In this approach (Fig. 1c), venom decomplexation is first performed by RP-HPLC on a C_18_ column at analytical scale, in the range of 0.5-2 mg of sample load. Resolved fractions are manually collected, and further separated by one-dimensional SDS-PAGE, where resulting protein bands can be excised and in-gel digested, to be finally submitted to MS/MS analysis. Comparatively, this approach is slow and requires significant manual work, especially in the collection and subsequent processing of chromatographic fractions. Furthermore, protein components that are present in trace amounts are generally more likely to be overlooked, in comparison to full LC-based strategies, due to the sampling bias of proteins that are more evident in the chromatographic pattern and the stained gels.

However, several advantages of this workflow may compensate these potential shortcomings, and altogether support its choice when the biological significance of the results is prioritized over the mere cataloguing of proteins:

- small peptides (or other compounds such as nucleosides) are recovered from the RP-HPLC step, in contrast to 2DE strategies;
- loading of the HPLC-resolved fractions onto gels for SDS-PAGE can be ‘normalized’ or adjusted, aiming to obtain protein bands of adequate staining-intensity (for in-gel digestion) even from chromatographic peaks that greatly differ in magnitude due to the dissimilar proportions of components in the venom. This normalization is not possible in the 2DE or LC-based shotgun workflows;
- analytical scale RP-HPLC allows for considerable venom sample loads, within the milligram range, which allows fractions to be recovered in sufficient amounts for complementary analyses, both functional and immunological, as will be discussed in the following sections;
- the relative abundances of identified proteins can be estimated from the integration of peak areas of absorbance at 215 nm (absorption wavelength of peptide bonds) in the RP-HPLC step, combined with densitometry scanning of the SDS-PAGE step when a fraction is resolved into several electrophoretic bands; and
- by performing SDS-PAGE of venom fractions under both reducing and non-reducing conditions, covalently-linked subunit composition of multimeric proteins can be deduced.

Regarding the basic equipment for sample decomplexation, the venomics strategy requires commonly available electrophoresis setup for SDS-PAGE (one dimensional), as opposed to higher cost isoelectrofocusing equipment needed for 2DE. It also requires regular HPLC instruments of analytical scale, in contrast to shotgun LC-based strategies which generally use more costly multidimensional nano-flow HPLC chromatographs.

On the side of drawbacks, the venomics workflow involves a more manually-oriented benchwork, and trace components are more prone to escape detection, as already mentioned. In addition, it has been noted that some large proteins of low abundance in the venom (for example hyaluronidases), might be difficult to elute from the C_18_ HPLC columns, and thus could be overlooked in some cases. Also, although most small and medium-sized venom components can be recovered in a functional state from the RP-HPLC separation, a number of larger proteins/enzymes become denatured by the acetonitrile gradients used for the elution, and therefore lose their activities, as discussed below.

## ‘Snake venomics’ as a useful proteomic profiling workflow

**Fig. 2:**
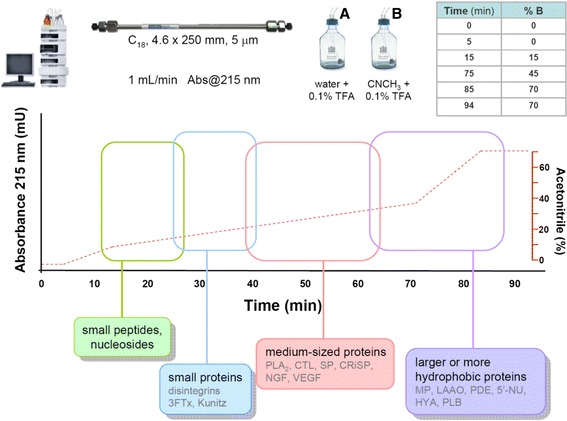
Scheme for RP-HPLC fractionation of snake venoms. A considerable number of snake venomic studies have used the chromatographic conditions indicated in the diagram. The venom proteins are separated using an analytical (4.6 × 250 mm, particle diameter of 5 μm) reverse-phase C_18_ column, eluted at a flow rate of 1 mL/min by a linear gradient of water containing 0.1% of trifluoroacetic acid (TFA) (solution A) and 70% acetonitrile (CNCH_3_) containing 0.1% TFAa, and the eluate monitored at 215 nm. The timetable for the mixing of these solutions (A, B), and the shape of the gradient (dashed line) are indicated. As an example, the approximate elution regions for some of the common protein components of snake venoms are indicated by colored boxes. This procedure has been applied to venoms of a number of viperid and elapid snakes, helping in the standardization and comparability of results between different laboratories. 3FTx: three-finger toxin; Kunitz: Kunitz-type serine protease inhibitor; PLA_2_: phospholipase A_2_; CTL: C-type lectin; SP: serine protease; CRiSP: cystein-rich secretory protein; NGF: nerve-growth factor; VEGF: vascular endothelium growth factor; MP: metalloproteinase; LAAO: L-amino acid oxidase; PDE: phosphodiesterase; 5′-NU: 5′-nucleotidase; HYA: hyaluronidase; PLB: phospholipase B

Currently, proteomic profiles of the venoms from more than 200 snake species have been reported in the literature, and numbers continue to grow. Venoms have been studied by a variety of analytical strategies, among them the ‘snake venomics’ workflow, utilized in the laboratories of both authors, has contributed with a considerable proportion of the published data. With the purpose of contributing to emerging research groups interested in this subject, a summary of the general conditions for the initial RP-HPLC separation of crude venoms used in many of the venomics studies is presented in Fig. 2.

The acetonitrile gradient used for elution (Fig. 2) is a scaled-down adaptation of the originally described method of 180 min [46] to 90 min [47], but retaining the same shape. A significant saving in time and solvents, without compromising resolution and pattern of elution, has been observed (unpublished results). Although each laboratory usually develops and optimizes its preferred HPLC protocols, adopting a common method could aid in the standardization and comparability of results among different research groups.

## Antivenomics: the immunorecognition profiling of venom antigens

An important area within snake venom research deals with the development, preclinical testing, and clinical monitoring of antivenoms used for the treatment of human or animal envenomation. These essential antidotes save thousands of lives every year. The preclinical characterization of antivenoms has mainly involved assays to assess their neutralizing potency against the lethal effect of whole venoms in animal models, usually mice, although often the neutralization of other relevant venom activities is reported as well [48].

**Fig. 3:**
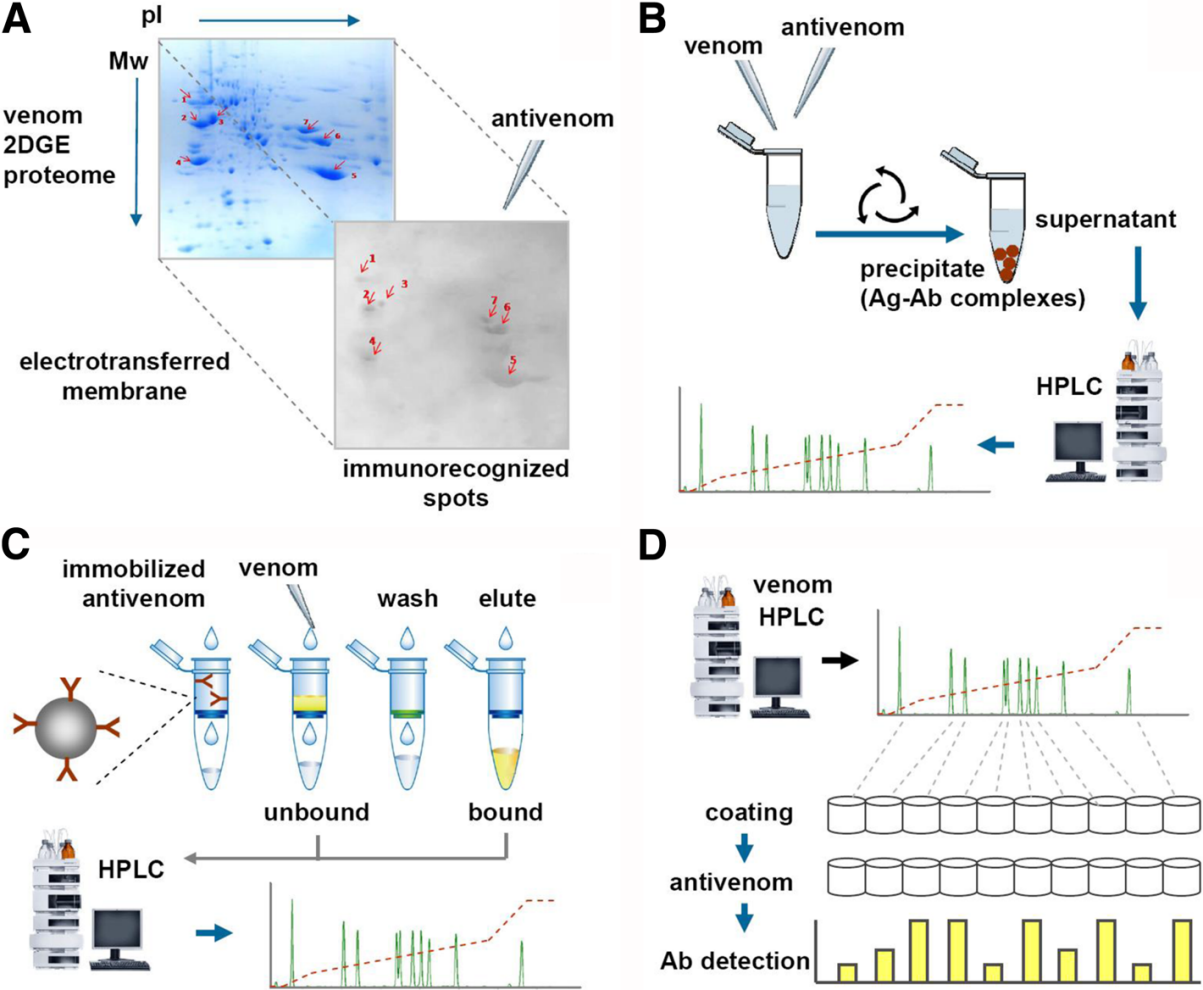
Antivenomic analytical strategies. A schematic representation of immunological approaches that have been combined with proteomic analysis of snake venoms, aiming to assess the immunorecognition of venom components by antibodies present in a given antivenom. **a** Immunoblotting, performed on electrotransferred membranes from two-dimensional gel electrophoresis (2DE) venom separations, identifies spots that are immunorecognized by the antivenom, in an essentially qualitative way. Immunoblotting can also be performed on membranes from the electrophoresis step (second dimension separation by SDS-PAGE) of the snake venomics strategy (see text and Fig. 1c). **b** ‘First generation’ antivenomics evaluates the immunodepletion of venom components after addition of antivenom and removal of precipitated immunocomplexes. The remaining supernatant is analyzed by HPLC and its profile is compared to that of a control venom aliquot. Differences in the chromatographic peaks between the antivenom-treated venom and the control venom can be quantified by integration of their peak areas, representing the immunodepletion of recognized components. **c** ‘Second generation’ antivenomics evaluates the venom components that are captured by an antivenom that has been covalently linked to beads, following the principles of immunoaffinity chromatography. Whole venom is incubated with this matrix and the unbound components are collected. After washing out the non-binding venom components, a change in pH elutes the bound venom fraction. Both samples are finally analyzed by HPLC, and their profiles are compared to that of a control sample of venom. Quantitative estimations of the degree of immunorecognition of components are performed as described for panel **b** by integration of chromatographic peak areas [58]. **d** HPLC/ELISA-based assessment of immunorecognition of venom components by an antivenom, or HPLC/ELISA-based immunoprofiling, is performed by coating microwell plates with a normalized amount of venom fractions obtained from the HPLC profile of the venom. Then, antivenom is added to each well and the bound antibodies (Ab) are detected by conventional ELISA

The introduction of proteomic analyses applied to snake venoms has opened new opportunities to deepen our knowledge on the detailed immunorecognition of venom components by antivenoms, an area that has been referred to as ‘antivenomics’ [49]. Taking advantage of the thorough compositional information on venoms provided by proteomic tools, methods have been devised to assess their individual component recognition by antibodies, using a variety of immunoassays (Fig. 3).

Antivenomic analyses can reveal which venom proteins are strongly, poorly, or even not immunorecognized by a given antivenom, providing valuable knowledge on the relative immunogenicity of these components in the animal species in which the antidote was produced. Moreover, these methods also offer a means for assessing cross-recognition between particular components in the venoms of different snake species, or intraspecific variations related to geographical distribution or age [32, 50–62]. In conjunction with venomics data, antivenomics represents a significant step forward in the preclinical characterization of antivenoms, bringing further information to support decisions on the selection of venom immunogens for the production of improved antivenoms, for example.

It must be stressed, however, that antivenomic analyses are restricted to the immunorecognition of venom antigens and, *sensu stricto*, this does not automatically imply neutralization of their toxic effects. For the purpose of the latter, neutralization assays remain the gold standard. Nevertheless, when dealing with polyclonal antibodies, immunorecognition is often a good predictor of neutralization. Therefore, antivenomic analyses provide highly valuable information to the overall characterization of antivenoms.

The original antivenomics protocol developed in Calvete’s laboratory [63] was based on the immunoprecipitation of antigen-antibody complexes formed by mixing of venom and antivenom in fluid-phase (Fig. 3b). Venom antigens are depleted from the supernatant if recognized by antibodies, and the RP-HPLC profile of the supernatant can then be compared to that of a control venom sample in order to assess the degree of immunodepletion of each peak. A second generation antivenomics protocol was developed (Fig. 3c), switching from a fluid-phase immunoprecipitation to a solid-phase interaction provided by immunoaffinity chromatography [64]. Antivenom is covalently immobilized onto the beads of an affinity matrix, which is then used to separate bound from unbound venom components. The antivenom-bound or ‘immunocaptured’ venom fraction is eluted by a change in pH, and then both fractions, as well as non-venom specific IgG and matrix controls, are analyzed by RP-HPLC to compare their profiles and quantify the degree of immunorecognition of each venom component.

Immunoaffinity-based antivenomic analyses require a careful control of all chromatographic conditions and a standardization of parameters for each particular antivenom/venom system. Inadequate proportions of venom and antivenom in the system might strongly affect the results due to the saturation of binding sites in the solid-phase matrix [65]. In addition, potential losses that may occur during the recovery of bound and unbound venom fractions must be taken into consideration to avoid introducing errors in the quantitative comparison of the subsequent HPLC profiles. On the other hand, the smoother baseline in chromatograms of the affinity column allowed better resolution and more accurate quantification of the antivenomic outcome than the original immunodepletion protocol. Furthermore, advantages of the second generation antivenomics are the possibility of analyzing F(ab’)_2_ antivenoms and the reusability of the affinity columns. These features contribute to the generalization, economy and reproducibility of the method.

The second-generation antivenomic strategy outlined above has been used most often in recent characterizations of antivenoms [66–68]. Additional types of immunoassays have also been combined with venomic analyses in order to evaluate the specificity of antibodies present in an antivenom toward particular venom proteins. Immunoblotting (Fig. 3a) can be performed on membranes electrotransferred from 2DE venom separations, incubated with antivenom, and developed for detection of bound antibodies [29, 34, 69]. In another immunoblotting strategy, the SDS-PAGE patterns of all venom fractions previously separated by RP-HPLC (following the ‘snake venomics’ protocol), can be electrotransferred and similarly developed with antivenoms [47, 63, 70–72]. Adequate parallel controls of non-immune sera matching the species from which antivenoms are produced are indispensable in all of these immunological techniques. Immunoblotting-based methods in the assessment of antivenom specificity have two important limitations: (a) results are essentially qualitative; and (b) some epitopes of venom components can be disrupted due to the denaturing effect of SDS detergent during either the 2DE or one-dimensional SDS-PAGE procedures.

A fourth approach for the antivenomic assessment of immunorecognition of venom components is based on enzyme-immunoassays such as the ELISA format (Fig. 3d). Protein peaks resolved by the RP-HPLC step of the venomics protocol are collected, normalized for concentration, and coated onto microwell plates. Then, the presence of antibodies toward each chromatographic fraction, in a given antivenom, can be determined by ELISA [73–79]. Although this combined HPLC/ELISA immunoprofiling approach provides a general view of the immunorecognition/immunogenicity of the different venom components along its full chromatographic elution profile, it is also not exempt from limitations. Among these, epitopes of venom antigens may become potentially altered by the solid-phase coating. Also, the intensity of absorbance signals provided by different venom fractions are influenced by a number of factors, such as epitope density and antibody saturation, thus precluding the possibility to perform quantitative calculations, as done in immunoaffinity-based antivenomics.

**Fig. 4:**
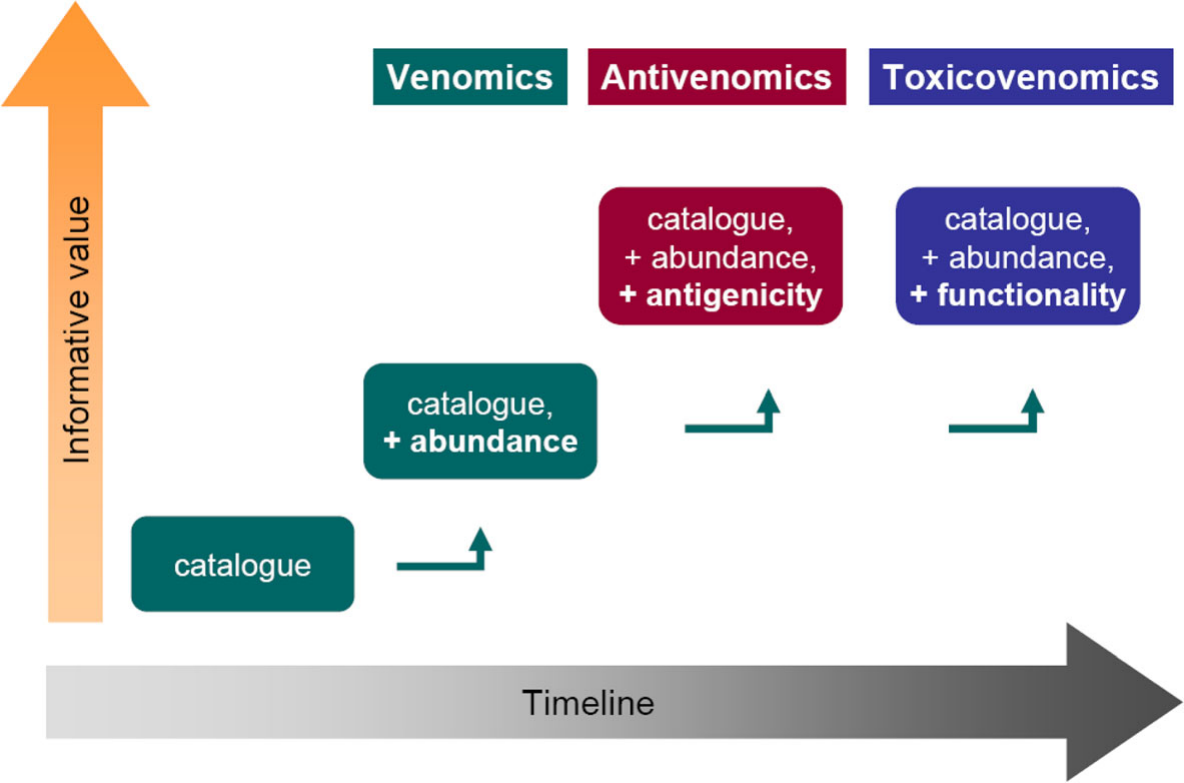
Evolution of analytical strategies in the characterization of snake venoms by proteomic tools, used in combination with appended methodologies. Initial proteomic studies on venoms essentially focused on the qualitative cataloguing of components. The introduction of the snake venomics strategy led to a valuable increase in the informative value of these analyses, by providing an estimation of the abundances of venom components. In combination with antivenomics, the immunogenicity of venom components can be inferred by evaluating their recognition by antibodies present in a given antivenom. A third dimension in the characterization of venoms is provided by a combination with toxicovenomics, which evaluates the toxic activities of components. Altogether, these combined strategies increase the informative value of studies characterizing venoms by disclosing their composition (venomics), immunorecognition (antivenomics) and toxicity (toxicovenomics)

Independently of the immunological methods adopted in the different analytical formats (Fig. 3), the possibility of combining the proteomic profile of venoms with the immunorecognition of its components by antivenoms, has provided a considerable increment in the informative value of studies in this field. By such combination of methods, information on antigenicity and immunorecognition can be added to the detailed cataloguing and abundance estimation of venom components (Fig. 4).

## Toxicovenomics: unmasking the villains among the crowd

Venoms are relatively complex secretions mainly composed of proteins and peptides which, by common sense, would be expected to display the major toxic activities of the venom. However, not necessarily every component present in a venom must be toxic, or not necessarily be toxic for every animal, whether experimental subject or natural prey. In addition, it seems reasonable to assume that some of the components may have a predominant role over others in the overall toxic effects of the venom. Recent studies have taken advantage of the known power of proteomic tools in dissecting and identifying the detailed composition of snake venoms, by combining this information with diverse functional assays (Fig. 4). Such combined strategy was first referred to as ‘toxicovenomics’ at the 18^th^ World Congress of the International Society on Toxinology (IST) held in Oxford in 2015 [80].

The essence of the toxicovenomics approach lies in screening the RP-HPLC resolved profile of venom fractions provided by the venomics workflow, for specific toxic activities. For example, screening for lethality to rodents would identify which venom components may play a role in the potentially lethal effects in humans, or screening for myotoxicity would identify components relevant to the skeletal muscle tissue damage induced by some venoms in clinical envenomation, and so forth. Thus, as a third pillar for a broader, more integrative view of snake venoms, toxicovenomic characterizations add valuable information of biological and medical significance.

A key concept related to toxicovenomic analysis was introduced by Laustsen et al. [81], which seeks to identify those components of a given venom that are mainly responsible for its toxicity, for example its lethal effects on mice: the ‘Toxicity Score’ (TS). By combining data on the identity, abundance, and lethal potency (median lethal dose; LD_50_) of each venom fraction, a TS is obtained by dividing its estimated relative abundance (% of total proteins) by its LD_50_ value. Then, it is possible to rank venom components in terms of their functional predominance to the overall effect of the venom, and therefore identify those that play most relevant roles.

The combination of toxic potency and abundance into a score allows a better view of the relevance of particular toxins in envenomation, as compared to toxic potency alone [81]. This concept was developed with the purpose of identifying which venom components should be targeted by novel neutralizing agents under development, such as recombinant human antibodies or synthetic peptide inhibitors [82]. Several investigations on elapid snake venoms have succeeded in pinpointing the main targets to be inhibited by using this experimental ‘toxicovenomics’ approach [73, 74, 78, 79].

Recent studies on the proteomic characterization of venoms are increasingly combining identification data with functional assays of particular components, to gain deeper insights from the medical and biological perspectives [57, 83–85]. The TS is conceptually identical to the ‘lethal neurotoxicity coefficient’ (LNC) defined as the ratio between the average LD_50_ and the crotoxin + crotamine relative abundance (% of the total venom proteins) [50]. The LNC was introduced to provide a quantitative measure of the evolutionary pressure towards gain of neurotoxicity and lethal activities of the venom of *C. durissus* snakes towards rodents, which evolved along the North-South axis of the invasion of South America, coincident with the evolutionary dispersal pattern of the Neotropical rattlesnakes [50]. This underscores the view that toxins bearing the highest toxicity score may represent the same proteins responsible for the evolutionary adaptive potential of venom. Hence, the toxicovenomic characterization of a venom is also of great relevance in the field of the evolutionary ecology of the organisms that produce the venom; and vice versa, the identification of toxins bearing the highest evolutionary pressure is also of great relevance for the design of more effective antidotes.

Although the addition of toxicovenomic evaluations to proteomic data appears in principle a simple concept, in practice there are still several important limitations to overcome. Among these is the fact that medium- to large-size enzymes/proteins may easily become denatured by the RP-HPLC conditions used to separate venoms. Metalloproteinases, for example, are inactivated by organic solvents commonly used in reversed-phase chromatography, and this has largely precluded the application of toxicovenomic strategies based on RP-HPLC to the venoms from viperids, which are generally rich in such enzymes. In the case of elapids, since many of them have very low proportions of metalloproteinases (i.e., < 5% of the total proteome), toxicovenomic screenings have succeeded owing to the fact that their major components, such as three-finger toxins, phospholipases A_2_, Kunitz-type serine protease inhibitors, etc., withstand the organic solvents and retain full functionality. However, there is a need to develop better-suited chromatographic methods under native conditions, using aqueous buffers, with a resolution capable of paralleling that of RP-HPLC, in order to expand the applicability of functional screenings to the venoms of viperids.

The resolution of size-exclusion chromatography (SEC)-HPLC columns is still comparatively low, and the use of ion-exchange HPLC-based columns limits the possibility to separate all venom components (acidic and basic) in a single run. Possibilities to combine different non-denaturing HPLC-based separations need to be explored in order to expand the applicability of toxicovenomic assessments to a broader range of snake species.

A second consideration about toxicovenomic evaluations concerns the possibility of having different venom components that act synergistically, i.e. where each of them separately may be weakly toxic, but together may result in a strong enhancement of a toxic effect, as identified, per instance, in *Micropechis ikaheka* venom [86]. Venoms whose sum of TS values of all separated fractions results in a significantly lower value in comparison to the TS of the unseparated material, should be suspected to enclose synergistic components [81].

A final consideration on toxicovenomic assessments relates to the choice of model for the evaluation of toxicity. It is known that some venoms may be highly toxic to certain types of animals, but not to others, and the concept of ‘taxon-specific toxins’ has been demonstrated in various studies [87–89]. As a general rule, experiments evaluating toxic activities with the purpose of investigating biological aspects, such as evolutionary or ecological inquiries, should consider the use of species reported to be natural prey for the particular venomous snake. Instead, for the study of applied aspects of venoms that are medically oriented, such as the development of antidotes or the study of pathological features experimentally induced by the toxins, mice or other mammalian models would be more pertinent, owing to their closer relatedness to humans and the ease of controlling all relevant variables to normalize the results.

## Conclusions

Undoubtedly, the application of proteomic tools to snake venom research has resulted in an unprecedented expansion of knowledge on their overall composition, in a growing number of species. Here, we have briefly discussed some recent developments in this area, highlighting how strategies have evolved from the mere cataloguing of venom components (proteomics/venomics), to a broader exploration of their immunological (antivenomics) and functional (toxicovenomics) characteristics (Fig. 4). Altogether, the combination of these complementary strategies is helping to build a broader view of the dangerous protein cocktails produced by venomous snakes, responsible for thousands of deaths every year around the globe. Such knowledge on snake venoms should provide better opportunities to cope with the great suffering inflicted on the individual and social levels [90, 91]. And, on the other hand, this knowledge should allow us to discover and explore the formidable bioactive molecules that venoms enclose, by developing beneficial applications, thus literally turning poisons into potions [92, 93].

Although it is hard to predict the future directions of a rapidly changing field dominated by technological advances – such as proteomics – it is likely that venomics will seek improved quantitative methods to calculate more accurately the abundance of venoms components [94]. Further, venomics will benefit from the rapidly increasing availability of genomic and transcriptomic data, to evolve its resolution power from a protein-family level, to a locus-resolution level, even encompassing proteoform variability [94]. Regarding antivenomics, the future should bring further refinements and application of techniques for determining the fine specificity of antibodies that recognize and neutralize toxins, identifying their most relevant antigenic determinants through strategies such as epitope mapping using sets of overlapping synthetic peptides [95–97], including the recently reported use of high-density peptide microarray technology for such purpose [98]. Toxicovenomics, still in its infancy, will need to cope with limitations and challenges already discussed, on the resolution of native chromatography strategies, and the development of pertinent bioassays, preferably in vitro.

Currently available methods in all these three areas that aim at an integrative view of the venoms are certainly not free of limitations and challenges. There is plenty of space for ingenious improvements, welcoming opportunities and ideas to develop and validate better procedures than the currently available. As earlier stated by the authors [99], a bright future for integrative venomics is on the toxinology horizon.

## Abbreviations

2DE: Two-dimensional gel electrophoresis
LC: Liquid chromatography
LD_50_: Median lethal dose
LNC: Lethal neurotoxicity coefficient
MS: Mass spectrometry
MS/MS: Tandem mass spectrometry
Mw: Molecular weight
pI: Isoelectric point
PTM: Post-translational modifications
SDS-PAGE: Sodium dodecyl sulfate-polyacrylamide gel electrophoresis
TFA: Trifluoroacetic acid
TS: Toxicity Score
